# Socioeconomic and environmental factors associated with dengue fever incidence in Guatemala: Rising temperatures increase dengue risk

**DOI:** 10.1371/journal.pone.0308271

**Published:** 2024-08-01

**Authors:** Kasem U. Salim, Francisco S. Álvarez, Alec M. Chan-Golston, Colleen C. Naughton, Ricardo Cisneros, Andrea Joyce

**Affiliations:** 1 Public Health, University of California Merced, Merced, CA, United States of America; 2 Fundación Naturaleza El Salvador, San Salvador, El Salvador; 3 Civil and Environmental Engineering, University of California Merced, Merced, CA, United States of America; Instituto Nacional de Salud Pública: Instituto Nacional de Salud Publica, MEXICO

## Abstract

Dengue fever is a mosquito-borne illness that infects 390 million people annually. Dengue outbreaks in Guatemala have been occurring more often and at increased rates since the first dengue outbreak in Guatemala in the 1970s. This study will examine environmental and socioeconomic factors associated with dengue in Guatemala at the municipality (county) level. Socioeconomic factors included population density, Mayan population, economic activity, and attending school. Environmental factors included average minimum annual temperature and annual precipitation. The relationship between environmental and socioeconomic variables and dengue fever incidence was initially evaluated through univariate zero-inflated negative binomial models, and then again through three zero-inflated multivariate negative binomial regression models. For all three models, elevation was considered a predictor of zero-inflation. In the combined model, there was a positive relationship between minimum temperature, economic activity and dengue fever incidence, and a negative relationship between population density, Mayan population and dengue fever. Predicted rates of dengue fever incidence and adjusted confidence intervals were calculated after increasing minimum yearly temperature by 1°C and 2°C. The three municipalities with the highest minimum yearly temperature (El Estor, Iztapa, and Panzós) and the municipality of Guatemala, all had an increase in the magnitude of the risk of dengue fever incidence following 1°C and 2°C increase in temperature. This research suggests that these socioeconomic and environmental factors are associated with risk of dengue in Guatemala. The predicted rates of dengue fever also highlight the potential effect that climate change in the form of increasing temperature can have on dengue in Guatemala.

## Introduction

### Global burden of dengue

Dengue fever is one of the most prevalent mosquito-borne diseases, and the reported cases have increased substantially over the past two decades [1]. In 2019, cases were reported from 129 countries. In addition, the actual number of dengue cases is underreported due to many asymptomatic or self-managed cases [1]. Bhatt et al. (2013) estimated 96 million dengue infections being diagnosed clinically and 390 million dengue infections per year [2]. Additionally, Zeng et al. (2021) approximated the number of deaths due to dengue fever has increased from 16,957 in 1990 to 40,467 in 2017, and there were 2,922,630 disability adjusted life years (DALYs) globally attributable to dengue in 2017 [3]. Although dengue fever incidence and deaths appear to have decreased for 2020 and 2021, COVID-19 most likely limited case reporting in many countries [1].

### *Aedes aegypti* and dengue

The mosquito *Ae*. *aegypti* is the primary vector of dengue virus transmission to humans [4, 5]. *Ae*. *aegypti* favor tropical and subtropical areas of the world; however, *Ae*. *aegypti* distribution could increase over time as climate change will result in higher global temperatures and lead to currently unfavorable areas being slowly transformed into areas more suitable for mosquito development [6].

Dengue virus (DENV) is characterized by four serotypes, DENV 1–4 [7]. When a person is bit by an *Ae*. *aegypti* mosquito and infected with dengue, they can develop mild or severe symptoms. Mild symptoms include fever, aches and pains, nausea, vomiting, and/or a rash which can be confused for the symptoms of common illnesses [8]. Once an individual has had dengue, infection with a different strain can result in severe dengue, also called dengue hemorrhagic fever. Dengue hemorrhagic fever can result in life-threatening symptoms only a few hours after infection and requires hospitalization [8].

### Climate and dengue

Naish et al (2014) conducted a systematic review of dengue and found that climate change will result in increased climatic suitability for dengue transmission as well as a geographical expansion of the regions at risk. Factors associated with climate change, such as increasing temperatures, rainfall and humidity could increase the rate of mosquito development, reduce virus incubation time, and create more breeding sites for *Ae*. *aegypti* [9]. *Ae*. *aegypti* distribution, and thus inherently dengue distribution, will expand as currently unfavorable areas are slowly transformed into more tropical or subtropical areas [6, 10]. Patz et al. (1998) examined the potential risk of increased disease transmission posed by climate change using computer-based simulation analysis. They found the largest increase in global epidemic potential in temperate regions. They also found that at increased temperatures, less mosquitoes will be needed to maintain dengue in regions where it is already endemic [11]. Tran et al. (2020) studied the potential threshold effects of climatic factors on dengue vector indices and found that an increase in 1°C in regions with an average warmer temperature (30.17°C) resulted in a larger increase in dengue infection rates than a 1°C increase in regions with an average colder temperature (27.21°C) [12].

### Factors associated with dengue in Guatemala

Guatemala is located in Central America. It is a small country, approximately the size of Kentucky in the United States [13], and it is located south of Mexico on the Pacific Coast. Generally, the terrain has a low-lying warm humid coastal zone, and interior highlands with altitudes up to 4,211 meters. Guatemala has a relatively large indigenous population (43.75%) which primarily identifies as Mayan [14]. Guatemala’s official language is Spanish with 69.9% of the Guatemalan population being Spanish speakers; however, 31% speak any one of a number of Mayan (29.7%), Xinca, and Garifuna (combined 0.4%) languages [14, 15].

Dengue is widespread in Central America including Guatemala [16]. The mosquito *Ae*. *aegypti* was considered eradicated from Guatemala in 1959 [17] but has been reintroduced. Dengue fever outbreaks first reemerged in Guatemala in the late 1970s [18]. The second outbreak did not occur until 1987 in the rainy season of Southern Guatemala’s Escuintla. Since that time, outbreaks have been occurring more often and at increased incidence rates with the largest dengue outbreak occurring in 2010 [16, 19]. Signor et al. (2020) studied 17 years of dengue fever surveillance data in Guatemala (2000 to 2016) in order to describe and identify epidemiological trends [16]. Several waves of dengue were identified, the largest peak occurring in 2010 and additional waves in 2003–2005 and 2012–2014. All four serotypes of dengue virus (DENV 1–4) were circulating in Guatemala, and more cases occurred during the rainy season from July through October [16]. Zika was additionally introduced into Guatemala by 2015 followed by Chikungunya, and both occurred along with dengue [20].

Socioeconomic risk factors in Guatemala, including rapid growth of the population density, increased population mobility, poverty, and lack of basic services, have contributed towards the spread of dengue’s epidemiological impact [19, 21]. Literacy and education have been associated with *Ae*. *aegypti* control practices, and school attendance has been found to impact dengue-related knowledge, attitudes, and practices [22–24]. Income has also been associated with *Ae*. *aegypti* with higher income areas having lower mosquito infestation rates and a potential lower risk of infection [25]. Having access to the internet contributes to quicker surveillance of infectious disease and rapid application of control strategies [26]. Another variable, urban population, has been positively associated with the number of dengue cases [27]. Additionally, homes without indoor plumbing can contribute to increased risk of dengue fever [28, 29], and population density has been associated with *Ae*. *aegypti* prevalence and dengue incidence [30–32]. Environmental risk factors have also been studied in Guatemala. Temperature and humidity are favorable for *Ae*. *aegypti* proliferation, and precipitation can be an effective predictor of dengue activity [19]. Other climatic factors that can affect dengue transmission in Central America are extreme anomaly phenomena such as El Niño Southern Oscillation (ENSO) [33].

The objectives of this study were to investigate socioeconomic and environmental variables associated with dengue fever incidence in Guatemala. Few studies have been conducted on dengue in Guatemala, yet there are typically tens of thousands of cases per year. We hypothesized that population density, economic activity, temperature, and precipitation would be positively associated with dengue cases, while school attendance and Mayan population would be negatively associated with dengue cases. We also hypothesized that modeling an increase in temperature of Guatemalan municipalities by 1°C- 2°C could increase dengue fever incidence in those municipalities.

## Materials and methods

### Ethics statement

This analysis used anonymous secondary sources of data collected for surveillance purposes and ethical approval was not required. No experimental work was undertaken outside of the analysis of anonymous secondary data.

### Socioeconomic and environmental variables

Municipality-level socioeconomic data were retrieved from the 2018 Guatemala census (XII Censo Nacional de Población y VII de Vivienda) produced by the Instituto Nacional de Estadistica Guatemala, available at ine.gob.gt [14]. The previous Guatemala census was conducted in 2002. In 2018, the population of Guatemala was 14,901,286. Guatemala is composed of 22 departments and 340 municipalities [14].

Socioeconomic variables considered for analysis were based on previous findings in the literature [22–32]. Variables included school attendance, economic activity, Mayan population, and population density. School attendance was defined as the percent of the population aged 7 or older that currently attend school. Economic activity was measured as the percent of the population aged 15 or older that are economically active. The Guatemala Census of 2018 did not contain an income variable; thus, the economically active population was chosen as a proxy for income. Population density was measured as the total population in a municipality divided by the total area (km^2^) of that municipality. Mayan population was defined as the percent of the population that identified as Mayan. The variable Mayan population was included because ethnic Mayan speak over 22 languages with different dialects which could make language barriers a challenge for health promotion and health services, as they may only target Spanish-speaking audiences [34, 35]. These variables were considered for inclusion in zero inflated negative binomial regression models, discussed below (Statistical Methods).

Environmental data were obtained from the Ministry of the Environment of Natural Resources [36]. Latitude and longitude of the county seat (the city that is the administrative center of the municipality), were obtained from Sistema Nacional de Información Territorial (SINIT) of the Secretaria General de Planificación y Programación de la Presidencia (SEGEPLAN). Data correspond to the location of each county seat (cabecera municipal). Environmental variables included the average minimum yearly temperature (°C), and total annual precipitation (mm), and elevation at the head of the municipality (county seat). Environmental variables were considered for analysis based on previous findings in the literature [32, 33]; these variables were also considered for inclusion in the regression models described below.

### Dengue cases

Total dengue cases for each of the 340 municipalities for 2017 and 2018 were obtained from the Ministry of Health of Guatemala (S1 Table). In 2015–2016, chikungunya and Zika were introduced into Guatemala and they were the focus of surveillance efforts; few dengue cases were reported in 2015–2016, relative to previous years. The years 2017–2018 were selected for analysis due to dengue case counts returning to levels similar to those reported before the introduction of chikungunya and Zika.

Data from 2017 and 2018 were combined for analysis. Data available for dengue cases was not distinguished as DENV 1–4. Cases were classified as mild (classic dengue) or severe (dengue hemorrhagic fever); only classic dengue were included. Dengue hemorrhagic fever (DHF) is extremely rare and individuals with DHF were not included due to low case counts. Cases were reported from small clinics and hospitals and made available for each municipality.

### Statistical methods

#### Calculation of incidence rate of dengue for each municipality

The incidence rate of dengue in each municipality was calculated. The number of dengue cases in the municipality for 2017–2018 combined was divided by the population in the municipality and the result was multiplied by 100,000. The rate is the cases per 100,000 people living in a municipality. The total dengue cases per year was also determined for 2017, 2018, and 2017–2018 combined for the entire country.

### Univariate zero-inflated negative binomial regression modeling

The socioeconomic and environmental variables described above were considered as predictors of dengue cases for regression models. Dengue cases are not normally distributed in municipalities; some municipalities have no or few cases and others have numerous cases. Dengue cases had a Poisson-like (right-skewed) distribution. While both Poisson and negative binomial regression models were initially considered, a likelihood ratio test detected significant evidence of overdispersion in the negative binomial model, suggesting that the negative binomial model was more appropriate than the Poisson model [37]. As a large number of municipalities (37.4%; 127/340) had zero incidences of dengue fever from 2017–2018, zero-inflated negative binomial (ZINB) models were also considered for analyses. Using base models without any covariates, the Akaike information criterion (AIC) and Bayesian information criterion (BIC) were compared between the negative binomial regression and the zero-inflated negative binomial regressions. As the AIC and BIC were lower for the zero-inflated negative binomial regression than for negative binomial regressions, ZINB regression was selected for analyses.

ZINB models were used to examine the relationship between the individual predictor socioeconomic and environmental variables (attending school, economically active, Mayan, population density, minimum temperature, and precipitation) and the rate of dengue cases per municipality (2017–2018) [37]. For all models, elevation was considered as a predictor of zero-inflation. The rationale behind this consideration was that higher elevations have cooler temperatures that do not meet the breeding requirements of *Ae*. *aegypti*, thus resulting in zero dengue cases in many of those municipalities.

In order to compare the 340 municipalities with differing populations, the socioeconomic count variables were first population standardized by dividing each observation by that municipality’s population. All covariates were then also converted to z-scores by subtracting the sample mean and dividing by the sample standard deviation for each variable. All analyses were implemented using Stata 17.0. Predictor variables with p-values less than 0.05 were considered significant.

### Modeling municipality rates of dengue: Zero-inflated negative binomial regression

ZINB multivariate models were used to assess if the rate of dengue cases per municipality (2017–2018) was associated with the socioeconomic or environmental variables above. Prior to running multivariate modes, all independent variables were examined for co-linearity to determine if any variables were significantly correlated. Zero-inflated negative binomial regressions were run separately for three groups of data, 1) socioeconomic variables, 2) the environmental variables, and 3) a combined model which included all the socioeconomic and environmental variables from the two models above. The socioeconomic variables included percent of the population attending school, percent of the population that are economically active, percent of the population that is Mayan, and population density. Environmental variables included average minimum annual temperature and total annual precipitation.

### Prediction values: Influence of temperature on predicted dengue cases

To examine the influence of temperature on predicted rates of dengue, seven representative municipalities were chosen to run predictions using a temperature increase in those municipalities by 1°C and 2°C. The seven municipalities included were the three municipalities with the lowest annual minimum temperature, the three with the highest minimum temperature, and the municipality of Guatemala, which contains the highly populated capital Guatemala City. Variables used in the previously mentioned combined model (Model 3) were included, which were percent attending school, percent economically active, percent Mayan, population density, temperature, precipitation, and elevation (as an inflate variable). The previously mentioned variables were all fixed using data in the original model for dengue cases 2017–2018. For each prediction interval, temperature was increased by 1°C and 2°C. The ideal temperature for *Ae*. *aegypti* survival has been identified as 20°C to 30°C [38], while other studies have specified steep increases in dengue incidence from 22°C to 29°C [39]. The three municipalities with the lowest minimum annual temperature, San Jose Ojetenam, Concepción Tutuapa, and Sibilia (4.78°C, 5.03°C, 5.10°C), all have average minimum temperatures far below the ranges of temperatures ideal for mosquito development, thus it is expected that the predicted rates of dengue fever incidence would be nonsignificant. The three municipalities with the highest minimum yearly temperatures were El Estor, Iztapa, and Panzós (22.56°C, 22.60°C, 22.78°C) which were all within the aforementioned 20°C to 30°C and 22°C to 29°C [39] temperature ranges predicted to be suitable for *Ae*. *aegypti* survival or an increase in dengue incidence. Thus, it was expected that 1°C and 2°C increases in temperature in municipalities with the highest minimum yearly temperature would likely lead to a higher predicted rate of dengue fever.

## Results

### Incidence rate of dengue in Guatemala (2017–2018)

**Fig 1:**
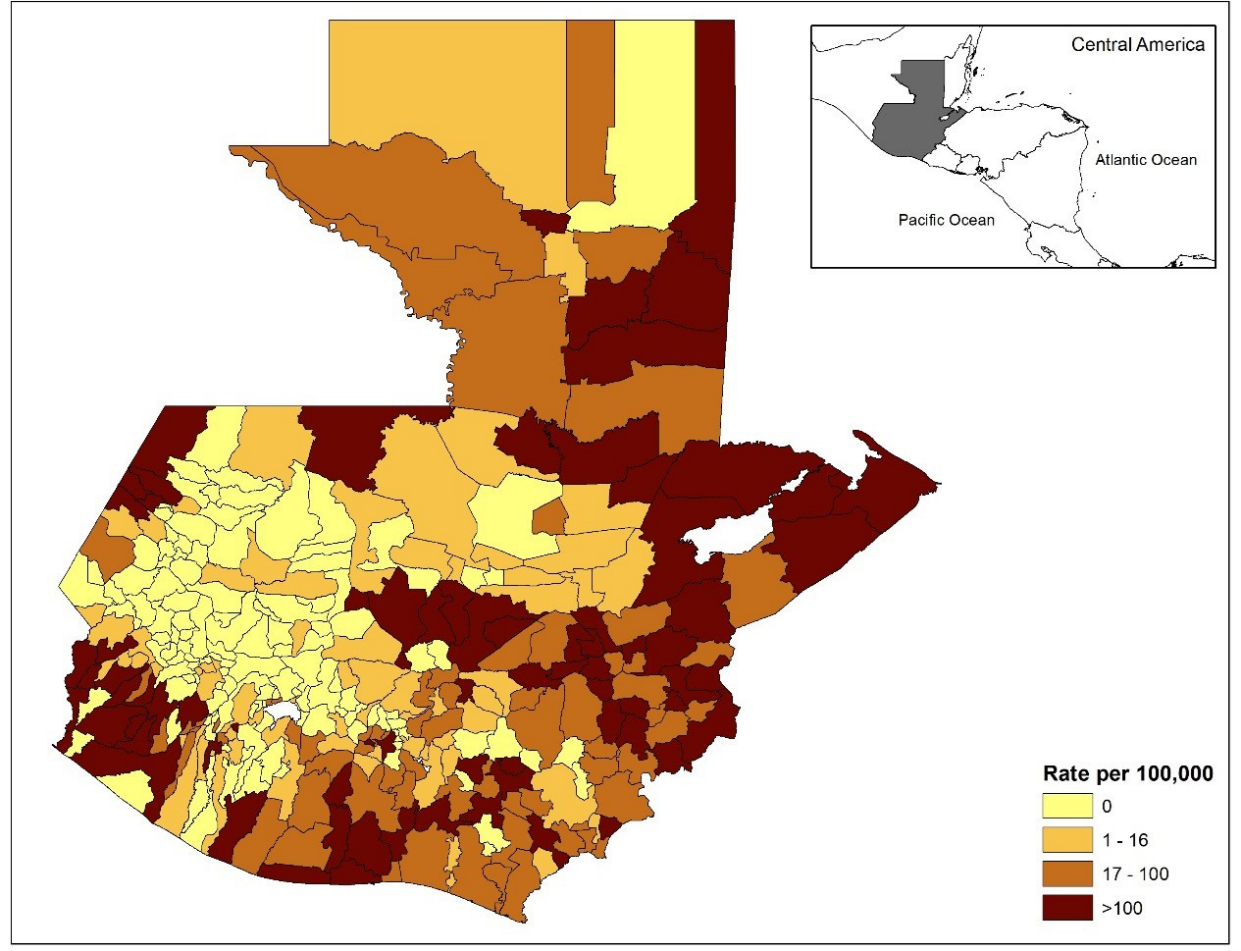
Dengue fever incidence rates per 100,000 people in Guatemala by municipality for the combined years of 2017 and 2018. Maps were made in ArcGIS 10.8 using Guatemala shape files available at the Secretariat of Planning and Programming of the Presidency (SEGEPLAN) of Guatemala, available at http://ide.segeplan.gob.gt/descargas.php.

**Fig 2:**
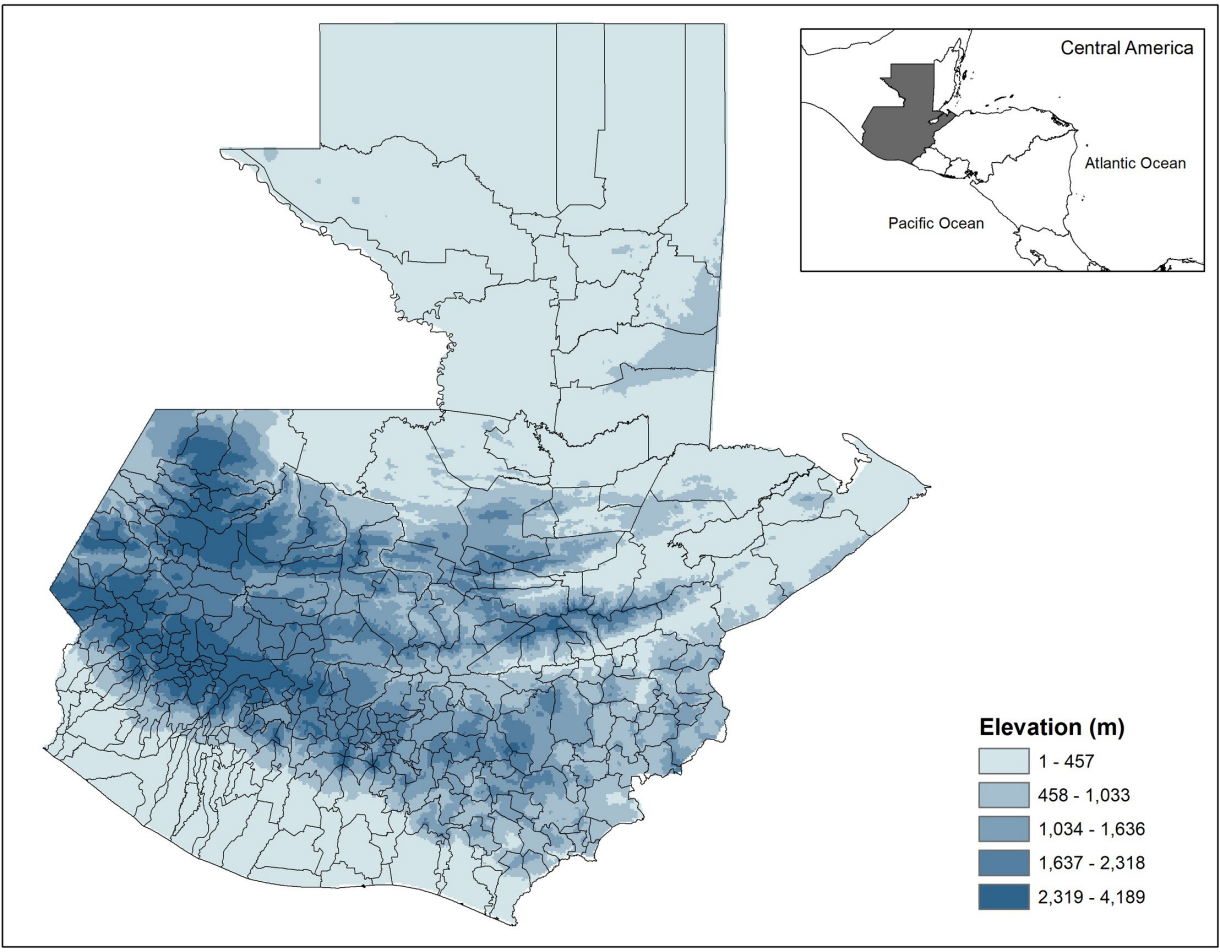
Elevation in municipalities in Guatemala. Darker coloration indicates higher elevation in the interior of the country. Maps were made in ArcGIS 10.8 using Guatemala shape files available at http://ide.segeplan.gob.gt/descargas.php.

**Fig 3:**
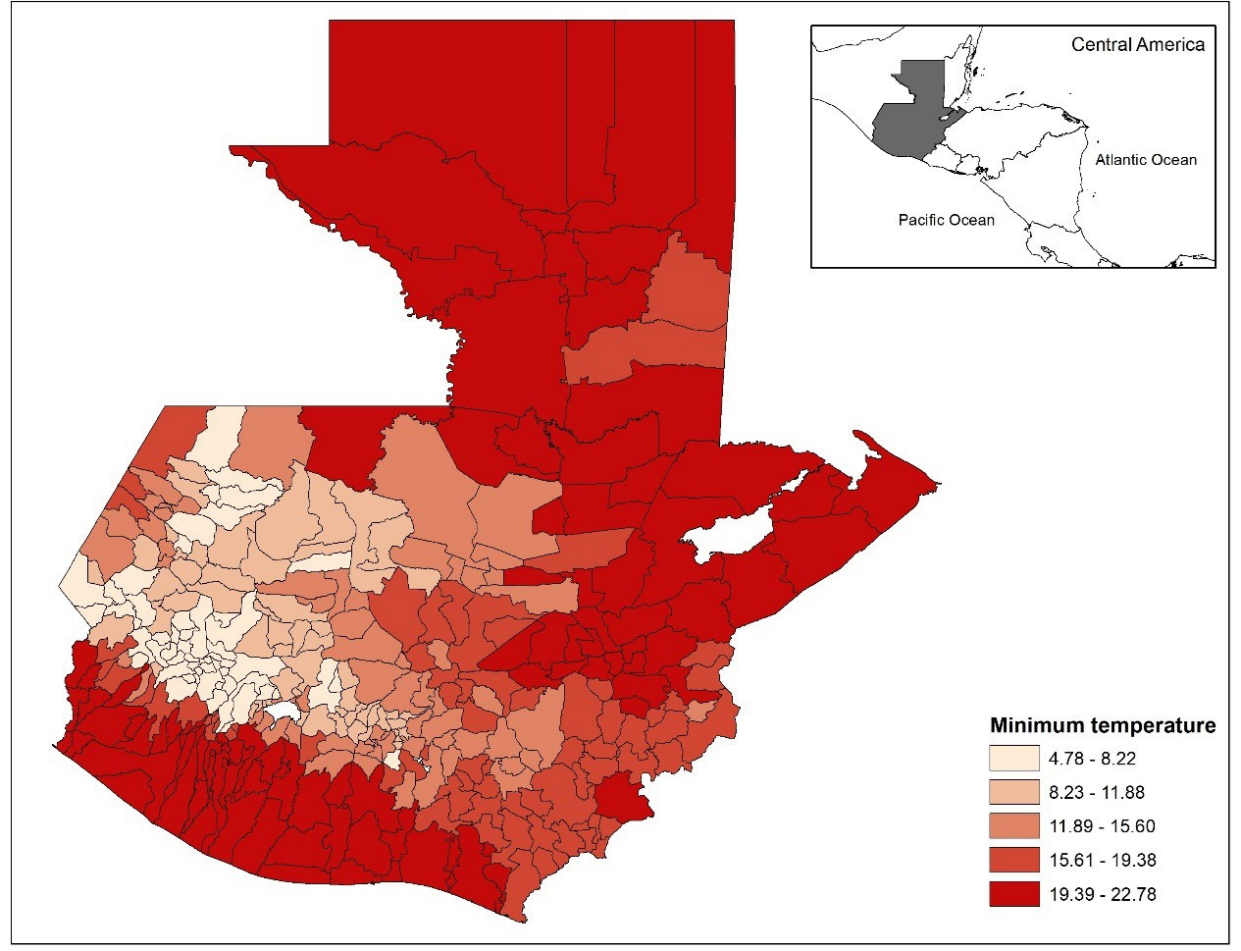
Minimum temperature (°C) in each municipality in Guatemala. Darker red indicates higher minimum annual temperature. Maps were made in ArcGIS 10.8 using Guatemala shape files available as described.

**Fig 4:**
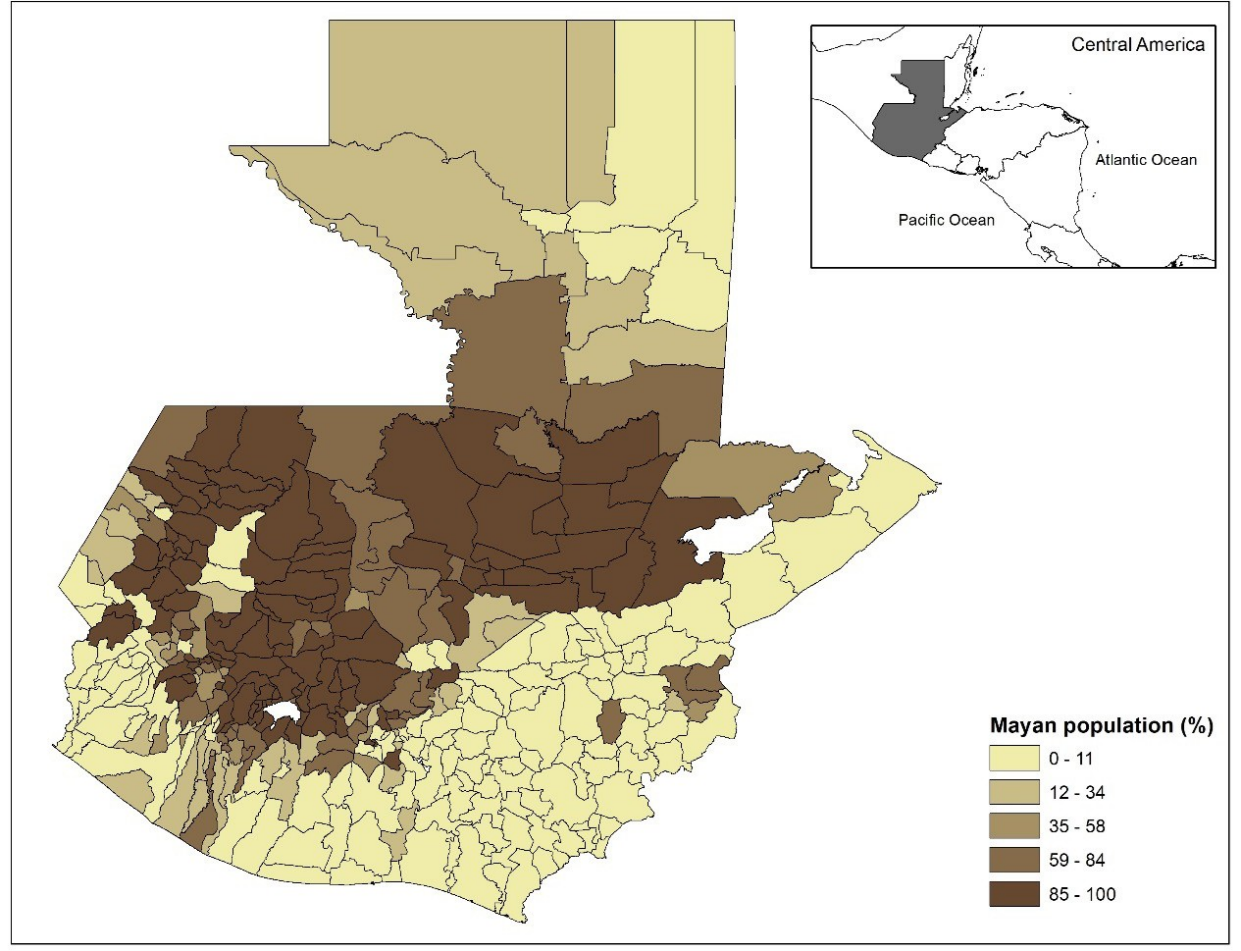
For each municipality in Guatemala, the percent of population which is Mayan. Maps were made in ArcGIS 10.8 using Guatemala shape files available at the Secretariat of Planning and Programming of the Presidency (SEGEPLAN) of Guatemala, available at http://ide.segeplan.gob.gt/descargas.php.

There were a total of 4,210 dengue cases for the year of 2017, and 7,414 dengue cases for the year of 2018, with a total of 11,624 dengue cases for the two-year combined period in Guatemala. The incidence rate of dengue in the municipalities of Guatemala for the two-year period (2017–2018) ranges from a low of 0 cases/ 100,000 to a high of 1,923 cases/ 100,000 people. The municipalities with higher incidence rates were more common on the periphery of the country, much of which has lower elevations (Figs 1 and 2). The inland portion of the country has higher elevation (Fig 2), lower minimum temperatures (Fig 3), and a higher percent of Mayan population (Fig 4). The municipalities with the top 10 incidence rates are shown in Table 1 alongside their population densities.

**Table 1:** Guatemalan municipalities with highest incidence rates of dengue for 2017–2018 / 100,000 residents.

Municipality	Incidence Rate per 100,000	Population Density
Coatepeque	1923.82	251.47
Genova	1245.43	222.42
Teculutan	931.71	83.04
Cabanas	872.37	98.42
Antigua Guatemala	790.38	667.59
Catarina	716.33	368.12
Zacapa	691.78	147.07
Guastatoya	680.88	113.81
Pachalum	644.87	211.27

The univariate zero-inflated negative binomial regressions of socioeconomic variables revealed a statistically significant negative association with population density (IRR = 0.75, 95% CI: 0.65, 086), and Mayan population (IRR = 0.51, 95% CI: 0.41, 0.65) (Table 2). The variables which were not significant were attending school and economically active. For environmental variables, minimum yearly temperature had a significant positive association with dengue cases (IRR = 3.86, 95% CI: 2.84, 5.28). Mean total precipitation was not significant (Table 2).

**Table 2:** Univariate zero-inflated negative binomial regression models including socioeconomic and environmental variables, and incidence rate ratios of the dengue cases for each municipality (2017–2018). IRR = Incidence rate ratio, CI = Confidence interval. N = 340 municipalities; * p<0.05.

Variables	IRR (95% CI)
Population Density	0.75 (0.65, 0.86) *
% Attending School	0.84 (0.64, 1.12)
% Economically Active	1.06 (0.82, 1.38)
% Mayan	0.51 (0.41, 0.65) *
Min Temperature	3.86 (2.84, 5.28) *
Precipitation	1.01 (0.86, 1.18)

### Zero-inflated multivariate negative binomial regressions

The zero-inflated multivariate negative binomial regression model of socioeconomic variables found that population density (p<0.001) and percent Mayan population (p<0.001) were significantly associated with the rate of dengue fever (2017–2018) (Table 3). The percent Mayan population had a negative relationship with the incidence rate ratio; an increase of one standard deviation (SD) Mayan population decreased the risk of dengue fever incidence by a factor of 0.52 (Table 3). Similarly, population density had a negative relationship with the incidence rate ratio; an increase of one standard deviation (SD) in population density decreased the risk of dengue fever incidence by a factor of 0.72 (Table 3). For the environmental model, temperature (p = 0.001) was a strong, significantly positive predictor of the rate of dengue fever cases (Table 4), while precipitation was not significant (Table 4)

**Table 3:** Socioeconomic variables included in the zero-inflated negative binomial regression model predicting incidence rate ratios of the dengue cases of each municipality in 2017–2018 (N = 340). IRR = Incidence rate ratio, CI = Confidence interval, OR = Odds Ratio. * p < 0.05.

Variables	IRR (95% CI)
Population Density	0.72 (0.61, 0.85) *
% Attending School	0.89 (0.67, 1.18
% Economically Active	1.11 (0.86, 1.44)
% Mayan	0.52 (0.41, 0.65) *
Intercept	0.0007 (0.0006, 0.0009) *
**Inflate**	**OR (95% CI)**
Elevation	22.66 (8.20, 62.61) *
Intercept	0.06 (0.02, 0.18) *
**Model Fit**	**Coefficient**
Alpha	2.43 (2.00, 2.92)

**Table 4:** Zero-inflated negative binomial regression model for environmental variables predicting incidence rate ratios of the dengue cases of each municipality in 2017–2018 (N = 340). * p < 0.05.

Variables	IRR (95% CI)
Min Annual Temperature	4.13 (2.95, 5.78) *
Precipitation	0.89 (0.75, 1.06)
Intercept	0.0004 (0.0003, 0.0006) *
**Inflate**	**OR (95% CI)**
Elevation	19.80 (5.94, 66.01) *
Intercept	0.04 (0.01, 0.16) *
**Model Fit**	**Coefficient**
Alpha	2.41 (2.00, 2.91)

The combined model for socioeconomic and environmental variables main effects found that minimum annual temperature (p< 0.001) and economic activity (p = 0.002) were significant positive predictors of dengue, while population density (p = 0.02) and Mayan population (p<0.001) were significant and were negatively associated with the rate of dengue fever cases (Table 5).

**Table 5:** Zero-inflated negative binomial regression model with combined socioeconomic and environmental variables, and incidence rate ratios of the dengue cases for each municipality (2017–2018). N = 340 municipalities; * p<0.05.

Variables	IRR (95% CI)
Population Density	0.82 (0.68, 0.98) *
% Attending School	0.86 (0.68, 1.10)
% Economically Active	1.53 (1.21, 1.94) *
% Mayan	0.64 (0.51, 0.81) *
Min Temperature	3.70 (2.61, 5.24) *
Precipitation	1.04 (0.87, 1.25)
Intercept	0.0003 (0.0002, 0.0004) *
**Inflate**	**OR (95% CI)**
Elevation	16.81 (4.34, 65.02) *
Intercept	0.032 (0.006, 0.17) *
**Model Fit**	**Coefficient**
Alpha	2.26 (1.87, 2.74)

Several factors increased the risk of dengue fever incidence. The risk of dengue incidence in a municipality increased by a factor of 3.70 for every one SD increase in minimum yearly temperature. An increase of one SD in the percent of economically active people in a municipality increased the risk of dengue fever incidence by a factor of 1.53. In addition, several factors decreased the risk of dengue. An increase of one SD in population density in a municipality reduced the risk of dengue by a factor of 0.82. Finally, for every SD increase in the percent of Mayan population, the risk of dengue fever incidence in that municipality was reduced by a factor of 0.64.

### Predicted values and adjusted confidence intervals

Predicted rates of dengue and corresponding confidence intervals were calculated for seven representative municipalities of the final model; this was done by increasing the temperature of those municipalities (Table 6). Predicted values and their corresponding confidence intervals were first calculated for when the all the variables in the combined model (Table 5, including temperature) were fixed at their sample values (Table 6). The municipalities with the lowest minimum temperatures, San Jose Ojetenam, Concepción Tutuapa, and Sibilia all had an incidence rate ratio (IRR) of 0 for dengue fever for the fixed temperature scenario. Subsequently, there was a less than or near 1 IR after the temperature adjustments (Table 6).

**Table 6:** Predicted rates of dengue fever (DF) incidence rate with an increase in temperature of 1–2°C for the three municipalities with the lowest minimum annual temperature, three with the highest minimum temperature, and for the municipality of Guatemala, which contains the capital Guatemala City.

Municipality	Annual Min. Temp	DF IR* per 100,000 with Fixed Temperature IR (95% CI)	DF IR per 100,000 After 1°C Increase IR (95% CI)	DF IR per 100,000 After 2°C Increase IR (95% CI)
Concepción Tutuapa	4.78°C	0.04 (-0.04, 0.12)	0.05 (-0.05, 0.16)	0.07 (-0.06, 0.20)
San Jose Ojetenam	5.03°C	0.16 (-0.25, 0.56)	0.20 (-0.32, 0.72)	0.26 (-0.40, 0.91)
Sibilia	5.10°C	0.57 (-0.23, 1.40)	0.73 (-0.29, 1.75)	0.93 (-0.36, 2.22)
El Estor	22.56°C	109.80 (47.86, 171.74)	140.34 (55.12, 225.56)	179.38 (62.03, 296.73)
Iztapa	22.60°C	349.17 (193.13, 505.21)	446.29 (227.85, 664.73)	570.43 (264.32, 876.54)
Panzós	22.78°C	104.35 (46.63, 162.07)	133.38 (53.61, 213.15)	170.47 (60.28, 280.66)
Guatemala	14.44°C	20.74 (0.63, 46.86)	30.34 (0.43, 60.25)	38.78 (-0.07, 77.63)

*DF = Dengue fever, IR = incidence rate

For the three municipalities with the highest minimum temperature, El Estor, Iztapa and Panzós, an increase in temperature did lead to a prediction of increased cases of dengue fever. El Estor, Iztapa, and Panzós had dengue fever IRs of 109.80, 349.17, and 104.35, respectively for the fixed temperature scenario and had higher rates of dengue fever after increasing temperature by 1°C (140.34, 446.29, 133.38), which increased further after increasing the temperature by 2°C (179.38, 570.43, 170.47). Similarly, the municipality of Guatemala had a dengue fever IR of 20.74 in the fixed temperature prediction scenario and an increased dengue fever IR for the 1°C increase scenario (30.34) and even further for the 2°C prediction scenario (38.78).

## Discussion

This study contributed to understanding how environmental and socioeconomic factors influence the distribution of dengue fever cases in Guatemala. This was initially evaluated through univariate zero inflated negative binomial (ZINB) regressions for the environmental and socioeconomic variables and the dengue cases, and then again through three zero-inflated multivariate negative binomial regression models. Finally, we examined how an increase in temperature contributed to predicted dengue cases in municipalities with the lowest and highest minimum annual temperature. For the univariate ZINB models, there was a statistically significant positive association of minimum temperature with dengue fever incidence, and a negative association with population density and Mayan population.

The multivariate regression models similarly pointed to variables which were significant predictors of dengue fever. When all variables were combined, the significant variables which were positively associated with dengue included minimum temperature and economic activity, while the variables population density and Mayan population were negatively associated with dengue fever. Precipitation was found to not be significantly associated with dengue cases, which contrasts with previous studies [11, 19, 32].

A significant finding from our study is the association between population density and dengue fever incidence. Population density has often been found to be associated with dengue incidence in previous studies [30–32]. For example, Tsuzuki et al. (2009) found that premises with six or more residents had significantly higher odds of repeated *Ae*. *aegypti* infestation when compared to households with 1–3 residents [31]. However, the present study found the relationship between population density and dengue fever was negative. A negative relationship between population density and dengue has been explained by others as well. For example, a significantly negative association was found by Schmidt et al. (2011); population densities ranging between ∼3000 to 7000 people/km^2^ in Vietnam were prone to dengue outbreaks [30]. These population densities were usually attributable to villages and peri-urban areas that did not have an adequate piped tap water supply. In the present study in Guatemala, low population density municipalities might lack tap water and instead use water storage containers that could be breeding sites for *Ae*. *aegypti*. Another possibility is that the most densely populated areas are at medium or higher elevation, with cooler temperatures and less hospitable to mosquito development. For example, the capital Guatemala City is highly populated and at 1493 m (∼4900 ft) elevation, as is nearby Antigua Guatemala (1500m). Many of the high incidence areas on the map are on the coast at low elevation areas, in areas with low to moderate population density, and the areas with highest minimum temperature.

This is the first study to examine and find a significant relationship between percent Mayan population and the risk of dengue fever incidence at the municipality level in Guatemala. Mayan population had a negative relationship with dengue incidence. Ethnic Mayan speak over 22 languages with different dialects which could make language barriers a challenge for health promotion and health services, as they may only target Spanish-speaking audiences [35]. Multiple studies have found Spanish fluency to be a significant predictor of health services utilization in Guatemala [40, 41]. However, in contrast, percent Mayan had a protective effect, municipalities with a higher percentage of Mayan population had a lower incidence of dengue fever. The Mayan populations may also live in areas with cooler temperatures that are less suitable for mosquito development, at higher elevations (Figs 2 and 4).

An important significant finding of our study was the impact of temperature on dengue risk. There was a positive relationship between average minimum yearly temperature and dengue fever incidence in Guatemala from 2017–2018. This result complements studies which found temperature significantly associated with dengue vector reproduction and transmission [42–45]. Gómez Gómez et al. (2022) investigated the association between climatic factors and dengue fever in Asuncion through piecewise regression models and found that minimum temperature was positively associated with dengue cases when the temperature was less than 21.3°C and negatively associated with dengue when greater than 21.3 °C [46]. Singh et al. (2022) also evaluated the relationship between minimum temperature and dengue incidence; however, they adjusted their minimum temperature to a two-month lag and found that minimum temperature at a 2-month lag was the best predictor of dengue incidence in Delhi [47]. Additionally, many studies found that minimum temperature was more predictive for dengue risk than maximum or mean temperatures [48–50]. Our study was also able to determine that minimum annual temperature was a better fit for the zero-inflated negative binomial model when compared to maximum or mean annual temperature.

Furthermore, elevation was considered as a predictor of zero-inflation, thus was used as an inflate variable for the zero-inflated negative binomial regressions. Others have found that Guatemalan land areas with elevations above 2000 meters have been modeled to have *Ae*. *aegypti* in only 0.90% of their land areas [51]. In all zero-inflated negative binomial regression models elevation was significant as an inflate variable.

Additionally, the prediction scenarios in this study found that dengue fever rates could increase in Guatemala with even a modest 1°C-2°C increase in temperature. While climate change may have numerous environmental impacts, we wanted to estimate the impact that climate change in the form of rising temperature on dengue incidence in the municipalities of Guatemala; we found that the three municipalities with the highest minimum temperatures (El Estor, Iztapa, and Panzós) would have an increase in their rates of dengue incidence. The three municipalities with the lowest average minimum yearly temperatures had little to no change in the magnitude of the rate of dengue fever incidence. Guatemala municipality, which contains the capital Guatemala City, had a minimum temperature of 14.44°C, and the increase of 1–2 °C similarly resulted in higher incidence rates of dengue.

The ideal temperature for *Ae*. *aegypti* survival has been identified as 20°C to 30°C [38], while other studies have specified steep increases in dengue incidence from 22°C to 29°C [39]. San Jose Ojetenam, Concepción Tutuapa, and Sibilia all had minimum temperatures below the ideal ranges for mosquito development, so it is reasonable that there were no large changes in dengue incidence. Conversely, El Estor, Iztapa, and Panzós had substantial increases in the dengue incidence for the 1°C increase scenario and even higher IRs after the 2°C increase. Tran et al. (2020) conducted a similar study assessing the potential threshold effects of climatic factors on dengue vector indices which found that an increase in 1°C did result in an increase in dengue infection rates, though there were variations in the magnitude of the increase depending on the temperature of the region [12]. In some municipalities, that increase in temperature would be sufficient to improve the suitability for *Ae*. *aegypti*.

Findings in this study lend support to the United Nation’s Paris Agreement and the 27th Conference of the Parties to the United Nations Framework Convention on Climate Change [52, 53]. One of the primary goals of COP27 was to hold the increase in global temperature to under 2°C above pre-industrial levels and ideally to 1.5°C above pre-industrial levels. Our findings support the importance of limiting the increase in temperatures, since even slight increases in minimum yearly temperature at the municipality level will result in large increases in the magnitude of risk of dengue fever incidence.

The present study had some limitations. Income is a prominent socioeconomic factor linked with increased dengue fever incidence [54, 55]; however, income was not included in the Guatemala census of 2018 and thus is lacking in this study. However, we were able to include economic activity as a proxy for income. Elevation was used as the inflate variable as it was believed to be the variable most attributable to municipalities with zero dengue cases; it is possible that there is another variable not included in the model that is primarily responsible for the municipalities with zero dengue cases. The temperature data we had available was a yearly average minimum temperature for each municipality. However, monthly or weekly data could provide more insight for tracking changes and duration of dengue incidences. Finally, the dengue fever case data did not have serotype available, which might vary in relation to environmental or other factors. Future studies could include these additional variables.

This study contributes to understanding how environmental and socioeconomic factors interact to influence the distribution of dengue in Guatemala. Socioeconomic factors such as Mayan population, population density, and economic activity, as well as the environmental factor of average minimum yearly temperature, played a significant role in predicting risk of dengue incidence. Future studies should continue to explore these factors and explore additional variables which were limited and outside the scope of this study. This will contribute to more effective dengue surveillance and vector control in Guatemala and limit the incidence of dengue.

## Supporting information

S1 Table Number of dengue fever cases for 2017–2018 for each municipality in Guatemala. (XLSX)

## References

[1] WHO. World Health Organization. Dengue-Global Situation. December 21, 2023. Available from: https://www.who.int/emergencies/disease-outbreak-news/item/2023-DON498

[2] Bhatt S, Gething PW, Brady OJ, Messina JP, Farlow AW, Moyes CL, et al. The global distribution and burden of dengue. Nature. 2013;496, 504–507. doi: 10.1038/nature12060 23563266 PMC3651993

[3] Zeng Z, Zhan J, Chen L, Chen H, Cheng S. Global, regional, and national Dengue burden from 1990 to 2017: A systematic analysis based on the global burden of disease study 2017. Eclinical Medicine. 2021;32,100712. doi: 10.1016/j.eclinm.2020.100712 33681736 PMC7910667

[4] CDC. Centers for Disease Control and Prevention 2020. Life cycle of Aedes aegypti and Ae. Albopictus mosquitoes. Centers for Disease Control and Prevention. Available from: https://www.cdc.gov/mosquitoes/about/life-cycles/aedes.html

[5] Ramchurn SK, Moheeput K, Goorah SS. An analysis of a short-lived outbreak of Dengue fever in Mauritius. Euro Surveill. 2009;14, 19314. doi: 10.2807/ese.14.34.19314-en 19712647

[6] Khormi HM, Kumar L. Climate change and the potential global distribution of Aedes aegypti: spatial modelling using GIS and CLIMEX. Geospatial health. 2014;8, 405–415. doi: 10.4081/gh.2014.29 24893017

[7] Whitehead S, Blaney J, Durbin A, et al. Prospects for a dengue virus vaccine. Nat Rev Microbiol. 2007;5, 518–528. doi: 10.1038/nrmicro1690 17558424

[8] CDC. Centers for Disease Control and Prevention 2019. Dengue Diagnosis. Centers for Disease Control and Prevention. Available from: https://www.cdc.gov/Dengue/healthcare-providers/diagnosis.html.

[9] Naish S, Dale P, Mackenzie JS, McBride J, Mengersen K, Tong S. Climate change and Dengue: a critical and systematic review of quantitative modelling approaches. BMC infectious diseases. 2014;14, 167. doi: 10.1186/1471-2334-14-167 24669859 PMC3986908

[10] Hales S, de Wet N, Maindonald J, Woodward A. Potential effect of population and climate changes on global distribution of Dengue fever: an empirical model. Lancet. 2002;14, 830–4. doi: 10.1016/S0140-6736(02)09964-6 12243917

[11] Patz JA, Martens WJ, Focks DA, Jetten TH. Dengue fever epidemic potential as projected by general circulation models of global climate change. Environ Health Perspect. 1998;106, 147–53. doi: 10.1289/ehp.98106147 9452414 PMC1533051

[12] Tran BL, Tseng WC, Chen CC, Liao SY. Estimating the Threshold Effects of Climate on Dengue: A Case Study of Taiwan. International journal of environmental research and public health. 2020;17, 1392. doi: 10.3390/ijerph17041392 32098179 PMC7068348

[13] One World Nations Online. Guatemala ‐ Republic of Guatemala ‐ Country Profile ‐ Nations Online Project. One World Nations Online. Available from: https://www.nationsonline.org/onewor13uatemalaala.htm#:%7E:text=With%20an%20area%20of%20109%2C000,the%20US%20state%20of%20Kentucky.

[14] UNFPA. United Nations Population Fund. National Institute of Statistics (Guatemala). Guatemala Population and Housing Census 2018. Available from: http://ghdx.healthdata.org/record/guatemala-population-and-housing-census-2018

[15] CIA. Central Intelligence Agency. Guatemala. The World Factbook. Available from: https://www.cia.gov/the-world-factbook/countries/guatemala/.

[16] Castillo Signor L, Edwards T, Escobar LE, Mencos Y, Matope A, Castaneda-Guzman M, et al. Epidemiology of Dengue fever in Guatemala. PloS Neglected Tropical Diseases. 2020;14, e0008535. Available from: 10.1371/journal.pntd.0008535 32813703 PMC7458341

[17] PAHO & WHO. Pan American Health Organization & World Health Organization. Report on the Status of Aedes aegypti eradication in the Americas. 1973. Available from: https://iris.paho.org/bitstream/handle/10665.2/26287/49248.pdf?sequence=1&isAllowed=y

[18] Wilson, ME, Chen LH. Dengue in the Americas. WHO Regional Office for South-East Asia. 2002. Available from: https://apps.who.int/iris/handle/10665/163755

[19] Ponciano JA, Polanco W, Barrios M. Dengue outbreaks pattern in southern Guatemala. 2019. 10.36829/63CTS.v6i2.631

[20] Lamb M, Paniagua-Avila A, Zacarias A, Rojop N, Chacon A, Natrajan MS, et al. Repeated rapid active sampling surveys demonstrated a rapidly changing Zika seroprevalence among children in a rural dengue-endemic region in Southwest Guatemala during the Zika epidemic (2015–2016). American Journal of Tropical Medicine and Hygiene. 2022; 107, 1099–1106. doi: 10.4269/ajtmh.22-0399 36252798 PMC9709015

[21] Kuno G. Review of the factors modulating Dengue transmission. Epidemiologic Reviews. 1995;17, 321–335. doi: 10.1093/oxfordjournals.epirev.a036196 8654514

[22] Hairi F, Ong CH, Suhaimi A, Tsung TW, bin Anis Ahmad MA, Sundaraj C, et al. A knowledge, attitude and practices (KAP) study on dengue among selected rural communities in the Kuala Kangsar district. Asia-Pacific journal of public health. 2003;15, 37–43. doi: 10.1177/101053950301500107 14620496

[23] Diaz-Quijano FA, Martínez-Vega RA, Rodriguez-Morales AJ, et al. Association between the level of education and knowledge, attitudes and practices regarding Dengue in the Caribbean region of Colombia. BMC Public Health. 2018;18, 143. doi: 10.1186/s12889-018-5055-z 29338712 PMC5771071

[24] AhbiRami R, Zuharah WF. School-based health education for dengue control in Kelantan, Malaysia: Impact on knowledge, attitude and practice. PLoS Negl Trop Dis. 2020;14, e0008075. doi: 10.1371/journal.pntd.0008075 32218580 PMC7141698

[25] David MR, Lourenço-de-Oliveira R, Freitas RM. Container productivity, daily survival rates and dispersal of Aedes aegypti mosquitoes in a high-income Dengue epidemic neighborhood of Rio de Janeiro: presumed influence of differential urban structure on mosquito biology. Memórias do Instituto Oswaldo Cruz. 2009;104, 927–932. 10.1590/S0074-02762009000600019. 19876569

[26] Marques-Toledo CdA, Degener CM, Vinhal L, Coelho G, Meira W, et al. Dengue prediction by the web: Tweets are a useful tool for estimating and forecasting Dengue at country and city level. PLOS Neglected Tropical Diseases. 2017;11, e0005729. doi: 10.1371/journal.pntd.0005729 28719659 PMC5533462

[27] Kolimenakis A, Heinz S, Wilson ML, Winkler V, Yakob L, et al. The role of urbanization in the spread of Aedes mosquitoes and the diseases they transmit—A systematic review. PLOS Neglected Tropical Diseases. 2021;15, e0009631. 10.1371/journal.pntd.0009631 34499653 PMC8428665

[28] Trevino J, Haque U. Socio-economic predictors of Dengue fever at the municipality level in Mexico. 2020. Available from: https://unthsc-ir.tdl.org/handle/20.500.12503/30240

[29] Joyce AL, Alvarez FS, Hernandez E. Forest Coverage and Socioeconomic Factors Associated with Dengue in El Salvador, 2011–2013. Vector-borne and zoonotic diseases. 2021.21, 602–612. doi: 10.1089/vbz.2020.2685 34129393

[30] Schmidt WP, Suzuki M, Dinh Thiem V, White RG, Tsuzuki A, et al. Population Density, Water Supply, and the Risk of Dengue fever in Vietnam: Cohort Study and Spatial Analysis. PLOS Medicine. 2011;8, e1001082. doi: 10.1371/journal.pmed.1001082 21918642 PMC3168879

[31] Tsuzuki A, Vu TD, Higa Y, Nguyen TY, Takagi M. Effect of peridomestic environments on repeated infestation by preadult Aedes aegypti in urban premises in Nha Trang City, Vietnam. Am J Trop Med Hyg. 2009;81, 645–650. doi: 10.4269/ajtmh.2009.08-0175 19815880

[32] Kalra NL, Kaul SM, Rastogi RM. Prevalence of Aedes aegypti and Aedes albopictus-Vectors of Dengue Hemorrhagic Fever in North, North-East and Central India. WHO Regional Office for South-East Asia. 1997. Available from: https://apps.who.int/iris/handle/10665/148533

[33] Zambrano LI, Sevilla C, Reyes-Garcia SZ, Sierra M, Kafati R, Rodriguez-Morales AJ, et al. Potential impacts of climate variability on Dengue hemorrhagic fever in Honduras, 2010. Trop Biomed. 2012;29, 499–507. 10.3390/tropicalmed7100322 23202593

[34] Ahmad M, Malik A, Mahmood K. Dengue-Related Information Needs and Information-Seeking Behavior in Pakistan. Health Communication. 2023;38, 1168–1178. doi: 10.1080/10410236.2021.1996674 34747288

[35] Instituto Nacional de Estadística (Guatemala). Encuesta Nacional de Condiciones de Vida 2014, tomo I. Guatemala City: INE 2016. Available from: https://www.ine.gob.gt/sistema/uploads/2016/02/03/bWC7f6t7aSbEI4wmuExoNR0oScpSHKyB.pdf

[36] MARN (Ministerio de Medio Ambiente y Recursos Naturales). Inventario Nacional Forestal de El Salvador. MARN, San Salvador, El Salvador. 2018;426. Available from: http://cidoc.marn.gob.sv/documentos/inventario-nacional-de-bosques

[37] Sokal RR, Rohlf FJ. Biometry (second edition). Macmillan. 1981.

[38] Tun-Lin W, Burkot TR, Kay BH. Effects of temperature and larval diet on development 476 rates and survival of the Dengue vector Aedes aegypti in north Queensland, Australia. Med. 2000;477, 31–37. 10.1046/j.1365-2915.2000.00207.x 10759309

[39] Fan J, Wei W, Bai Z, Fan C, Li S, Liu Q, et al. A systematic review and meta-analysis of Dengue risk with temperature change. International journal of environmental research and public health. 2015;12(1): 1–15. 10.3390/ijerph120100001 PMC4306847 25546270

[40] Chomat AM, Solomons NW, Montenegro G, Crowley C, Bermudez OI. Maternal health and health-seeking behaviors among indigenous Mam mothers from Quetzaltenango, Guatemala. Rev Panam Salud Pública. 2014;35(2): 113–20. 24781092

[41] Ishida K, Stupp P, Turcios-Ruiz R, Williams D, Espinoza E. Ethnic Inequality in Guatemalan Women’s Use of Modern Reproductive Health Care. International Perspectives on Sexual and Reproductive Health. 2012;38(2): 99–108. doi: 10.1363/3809912 22832150

[42] Watts D, Burke D, Harrison B, Whitmire R, Nisalak A. Effect of temperature on the vector efficiency of Aedes aegypti for Dengue 2 virus. Am J Trop Med Hyg. 1987;36: 143–152. doi: 10.4269/ajtmh.1987.36.143 3812879

[43] Alto BW, Bettinardi D. Temperature and Dengue virus infection in mosquitoes: independent effects on the immature and adult stages. The American journal of tropical medicine and hygiene. 2013;88(3): 497–505. doi: 10.4269/ajtmh.12-0421 23382163 PMC3592531

[44] Jansen CC, Beebe NW. The Dengue vector Aedes aegypti: What comes next. Microbes Infect. 2010;12, 272–279. doi: 10.1016/j.micinf.2009.12.011 20096802

[45] Morin CW, Comrie AC, Ernst K. Climate and Dengue transmission: evidence and implications. Environmental health perspectives. 2013;121(11–12): 1264–1272. doi: 10.1289/ehp.1306556 24058050 PMC3855512

[46] Gómez Gómez RE, Kim J, Hong K, Jang JY, Kisiju T, Kim S, et al. Association between Climate Factors and Dengue fever in Asuncion, Paraguay: A Generalized Additive Model. Int. J. Environ. Res. Public Health. 2022;19: 12192. 10.3390/ijerph191912192 PMC9566529 36231491

[47] Singh PS, Chaturvedi HK. A retrospective study of environmental predictors of dengue in Delhi from 2015 to 2018 using the generalized linear model. Sci Rep 2022;12: 8109. doi: 10.1038/s41598-022-12164-x 35577838 PMC9109956

[48] Li Y, Dou Q, Lu Y, Xiang H, Yu X, Liu S. Effects of ambient temperature and precipitation on the risk of Dengue fever: A systematic review and updated meta-analysis. Environmental Research. 2020;191: 110043. doi: 10.1016/j.envres.2020.110043 32810500

[49] Yu HL, Lee CH, Chien LC. A spatiotemporal Dengue fever early warning model accounting for nonlinear associations with hydrological factors: a Bayesian maximum entropy approach. Stoch. Environ. Res. Risk Assess. 2016;30(8): 2127–2141.

[50] Cheong Y, Burkart K, Leitão P, Lakes T. Assessing weather effects on dengue disease in Malaysia. Int. J. Environ. Res. Publ. Health. 2013;10(12): 6319–6334. doi: 10.3390/ijerph10126319 24287855 PMC3881116

[51] Watts AG, Miniota J, Joseph HA, Brady OJ, Kraemer MU, Grills AW, et al. Elevation as a proxy for mosquito-borne Zika virus transmission in the Americas. PloS one. 2017;12(5): e0178211. doi: 10.1371/journal.pone.0178211 28542540 PMC5443570

[52] UN. The Paris Agreement: United Nations 2022. Available from: https://www.un.org/en/climatechange/paris-agreement.

[53] UN. 27th Conference of the Parties: United Nations. Available from: https://www.un.org/en/climatechange/cop27. Accessed December 10, 2022.

[54] Mulligan K, Dixon J, Sinn CL, Elliott SJ. Is Dengue a disease of poverty? A systematic review. Pathogens and global health. 2015;109(1): 10–18. doi: 10.1179/2047773214Y.0000000168 25546339 PMC4445289

[55] Lee JS, Mogasale V, Lim JK, Carabali M, Lee KS, Sirivichayakul C. A multi-country study of the economic burden of Dengue fever: Vietnam, Thailand, and Colombia. PLoS neglected tropical diseases. 2017;11(10): e0006037. doi: 10.1371/journal.pntd.0006037 29084220 PMC5679658

